# Preventive wound drainage reduces esophageal fistula or infection after endoscopic resection of giant submucosal tumors in the esophagus

**DOI:** 10.1055/a-2687-3086

**Published:** 2025-09-04

**Authors:** Qiao Yun Liao, Yi Meng Tang, Li Sha Zhan, Yao Fan

**Affiliations:** 1 613497 General Surgery, The Third Hospital of Mianyang, Mianyang, China

**Keywords:** Endoscopy Upper GI Tract, Endoscopic resection (ESD, EMRc, ...), Benign strictures, Dilation, injection, stenting

## Abstract

**Background and study aims:**

Submucosal tunneling endoscopic resection (STER) has emerged as an innovative approach for the treatment of giant submucosal tumors (SMTs) in the esophagus. However, complications such as esophageal fistula or submucosal infection remain a concern. This article explores how preventive wound drainage can play a significant role in reducing these complications.

**Patients and methods:**

We devised an innovative and straightforward method for negative pressure drainage. This approach involves positioning the drainage device with metal clips before closing the esophageal mucosa wound. A retrospective analysis was conducted on 46 patients with giant SMTs who underwent the STER procedure, among whom 28 had drainage and 18 had no drainage. Patient characteristics, adverse events, and risk factors were comprehensively evaluated.

**Results:**

In 46 patients, the transverse diameter of the tumor exceeded 5 cm. No significant difference was observed in age, gender, tumor size, surgical scope, or mucosal injury between the two groups studied ( *P* > 0.05). Esophageal fistula or submucosal infection rates in the drainage group were lower than those in the no drainage group (2/28 vs 14/18, *P* < 0.05). Subgroup analyses revealed that multiple injuries in the esophageal mucosa combined with full-thickness resection of the esophageal muscle layer were the immediate causes of esophageal fistula or submucosal infection following STER surgery.

**Conclusions:**

In the context of STER for giant esophageal submucosal tumors with muscular layer full-thickness resection and mucosal injury, preventive drainage is an effective strategy for minimizing postoperative esophageal fistula and submucosal infection complications.

## Introduction

Submucosal tumors (SMTs) of the gastrointestinal tract, including mesenchymomas, leiomyomas, lipomas, neurogenic tumors, fibromas, and hemangiomas, have been increasingly detected and diagnosed through endoscopic ultrasonography (EUS) 1 . The occurrence of SMTs in the upper gastrointestinal tract is predominantly observed in the esophagus and stomach. Among these, stromal tumors and leiomyomas constitute the most prevalent pathological types 2 . SMTs exhibit varying growth patterns depending on their location, with some tending to protrude inward toward the lumen, whereas others may extend outward from the cavity. Considering the potential for malignancy in SMTs, surgical resection, particularly endoscopic resection, is viewed as a pivotal therapeutic approach.

Submucosal tunneling endoscopic resection (STER), leveraging its advantage of preserving the integrity of the digestive tract mucosa, has emerged as a commonly employed endoscopic technique for managing submucosal tumors (SMTs) situated in the upper digestive tract 3 4 . Although STER has demonstrated its efficacy and safety as a treatment option for SMTs of the esophagus, it is worth noting that irregular tumor shapes and those with diameters exceeding 2.5 cm may pose a higher risk of postoperative complications, including bleeding, esophageal fistula or hematocele, and infection 5 6 . Previous research has indicated that in individuals who suffer from giant esophageal leiomyomas (with a transverse diameter ≥ 3 cm), the likelihood of developing an esophageal fistula during the procedure of STER is 6.25% 7 . in addition, we observed that as the transverse diameter of esophageal submucosal tumors increases, and particularly in patients who experience multiple mucosal injuries during surgery, risk of developing postoperative esophageal fistula and infection rises accordingly. However, esophageal fistula or infection remains the most severe complication following STER operation for esophageal submucosal tumors, notably prolonging the length of hospital stay. Unfortunately, at this juncture, there is a lack of a straightforward preventive measures to address this issue.

To address the challenge of reducing esophageal fistula and infection complications following STER procedure for removal of SMTs, we innovatively incorporated a step positioning the drainage device prior to closing the esophageal mucosa wound with metal clips. Interestingly, implementing this approach led to a remarkable reduction in esophageal infection complications among patients with giant SMTs who underwent STER surgery. This finding underscores the effectiveness and potential of our novel technique in enhancing postoperative recovery and patient safety.

## Patients and methods

### Patients

A retrospective study was conducted on 46 patients with large SMTs in the esophagus who underwent STER in two hospitals from January 2020 to April 2025, including Affiliated Zhongshan Hospital of Fudan University and The Third Hospital of MianYang. Preoperative EUS and contrast-enhanced chest computed tomography (CT) were utilized to confirm the origin of the SMTs from the muscularis propria layer, with the maximum transverse diameter of the tumors exceeding 5 cm. Prior to the operation, all patients were informed of the potential benefits and risks associated with the treatment. Written informed consent was obtained from each patient to ensure informed decision-making and ethical compliance.

### STER procedure

The following instruments and equipment were used for STER: standard single-channel gastroscope (GIF-Q290J; Olympus Optical Co. Ltd, Tokyo, Japan), transparent cap (D-201–11802; Olympus), Hybridknife (W0406468, Germany), IT knife (KD-620LR; Olympus), injection needle (NM-4L-1; Olympus), snare (SD-230U-20; Olympus), Coagrasper hemostatic forceps (FD-410LR; Olympus), Hemostatic clips (HX-600–135; Olympus; Resolution, Boston, Massachusetts, United States), CO _2_ insufflator (Olympus), and ERBE electrosurgical coagulation unit with high-frequency generator (VIO 200D; ERBE Elektromedizin GmbH, Tübingen, Germany), Nasogastric tube (Link-02–1, China).

All patients received general anesthesia via endotracheal intubation and were placed in the left lateral decubitus position. A transparent cap was attached to the distal end of the endoscope. An incision was made at the mucosa located approximately 5 cm proximal to the tumor. A mixed solution of epinephrine and indigo carmine diluted in 0.9% NaCl solution was used for submucosal injection to create a local fluid cushion in the mucosal layer. Then, a 2-cm mucosal incision was made longitudinally along the esophagus using a Hybridknife, and the endoscope was inserted through the incision site into the submucosa. The submucosa and muscularis propria layers were gradually separated using a Hybridknife. A longitudinal tunnel was created between the two layers until the tumor was clearly exposed in the tunnel. The tumor was completely dissected from the surrounding tissues with an intact capsule using a Hybridknife and IT knife. Tumor extraction was then performed several times using a snare. After tumor resection, all visible blood vessels were coagulated with hot biopsy forceps. Before closing the wound with metal clips, an extra step was incorporated. Specifically, a gastric tube with a 4.7-mm diameter was inserted beneath the wound mucosa to facilitate drainage. Subsequently, hemostatic clips were utilized to completely seal the tunnel, starting from the distal end and progressing toward the proximal end. At the proximal end of the wound, the gastric tube was then led out, serving as a crucial pathway for fluid removal and ensuring proper management of the post-procedure situation.

### Postoperative management

Postoperative medication included proton pump inhibitors and antibiotics, and dietary restriction was mandatory. Postoperative nursing interventions involved temperature monitoring and infusion. Daily blood routine examinations were carried out for 3 days after surgery. The drainage tube was maintained under continuous negative-pressure suction, and the color and properties of the drained fluid were observed. If the drainage fluid became turbid, it was necessary to send the drainage fluid for bacterial culture combined with drug-sensitivity testing. Procalcitonin test and chest CT imaging were performed on Days 2 and 3 post-STER, respectively. Gastroscopy examination was carried out 72 hours post-STER for the purpose of evaluating the wound. Simultaneously, patients with drainage tubes were recommended to have the tubes removed and the defects sealed with metal clips. In case of infection in the wound, all metal clips were removed and thoroughly rinsed for drainage.

### Data collection and statistical analysis

Collection of clinical records included gender, age, tumor site, pathology, and surgical scope. SPSS statistical software version 20.0 (IBM Corporation, United States) was used for data analysis. Descriptive statistics were used to summarize clinicopathological characteristics of the patients. Categorical variables were compared using the t test, χ2 test, and Fisher's exact test. The test of significance was a two-sided *P* < 0.05, and the difference was considered statistically significant.

## Results

### Clinical characteristics

Baseline characteristics of the 46 patients in this study are shown in Table 1 . The study was composed of 26 male and 20 female patients. No significant difference was observed in age, gender, tumor size, pathology results, surgical scope, or mucosal injury between the two groups studied ( *P* > 0.05). In the non-preventive drainage group, 12 patients suffered from submucosal infection and/or esophageal fistula; in contrast, in the preventive drainage group, only two cases of such problems occurred.

**Table:** **Table 1** Clinical characteristics and outcome of different strategy for giant submucosal tumors in the esophagus.

	**No drainage (n = 18)**	**Preventive drainage (n = 28)**	***P* value **
Age, mean (range), years	58.06 (22.00–78.00)	53.46 (17.00–78.00)	0.521
Gender (male/female)	10/8	16/12	0.382
Size, median (range), cm	5.50 (5.00–6.20)	5.72 (5.10–6.30)	0.278
Pathology results, n (%			
Leiomyoma	18 (100%)	28 (100%)	
Surgical scope, n (%)			
Submucosal layer	6 (33.33%)	10 (35.71%)	1.00
Muscularis propria layer	12 (66.67%)	18 (64.29%)	
Mucosal injury, n (%)	7 (38.89%)	10 (35.71%)	0.56
Submucosal infection and/or esophageal fistula n (%)	12 (66.67%)	2 (7.14%)	0.038

### Application of preventive wound drainage in the STER procedure

**Fig. 1:**
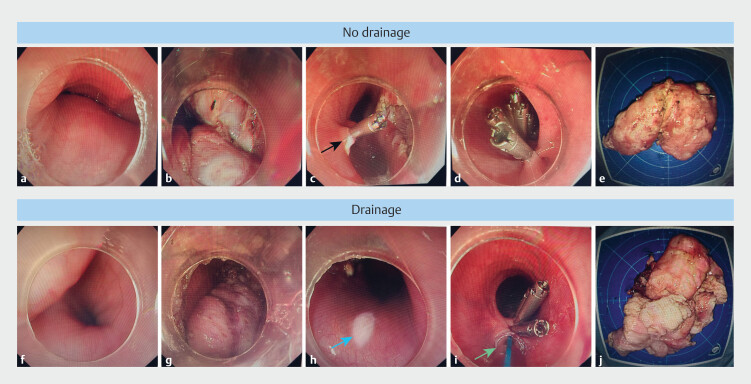
Different STER procedure for giant SMTs in the esophagus. **a, b, c, d, e** The giant submucosal tumor in the esophagus was removed through the traditional STER surgical method. The black arrow points to the mucosal damage. **f, g, h, i, j** The traditional STER surgical method combined with preventive wound drainage was employed for removal of the giant submucosal tumor in the esophagus. The blue arrow indicates mucosal damage. The green arrow shows the position of the drainage tube.

Esophageal fistula and infection following the STER procedure for removal of SMTs are indeed severe and concerning complications. To mitigate such complications, a preventive drainage strategy was devised. This strategy involves surgical steps similar to the original STER procedure. However, at the crucial stage of closing the tunnel wound at the end, a distinct step was added. Specifically, a negative-pressure drainage tube was placed under the mucosa in advance. This anticipatory strategy was expected to play a vital role in reducing the occurrence of esophageal fistula and infection. Fig. 1 shows an example of the STER procedure and drainage.

### Preventive wound drainage reduces esophageal fistula or infection after STER of giant submucosal tumors in the esophagus

**Fig. 2:**
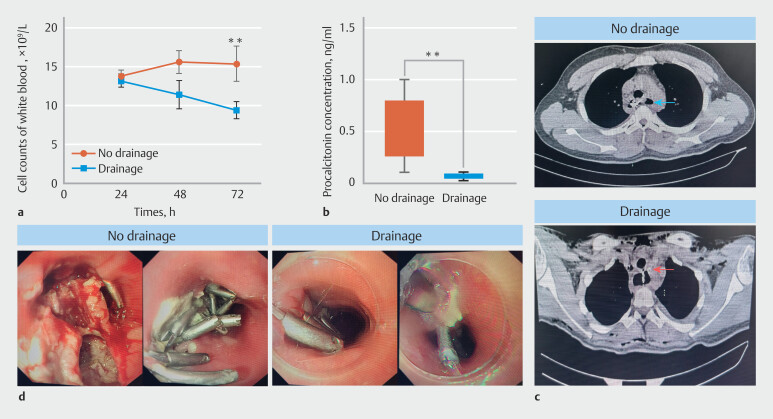
Preventive wound drainage reduces esophageal fistula or infection following STER procedure. **a** The white blood cell count was measured by blood routine test. ** *P* < 0.01, t test. **b** Serum procalcitonin was detected by Elisa test. ** *P* < 0.01, t test. **c** On the third day following the STER surgery, a chest CT scan was carried out to assess the condition of the esophagus and its surrounding regions. The blue arrow indicates fluid and gas accumulation under the esophageal wound. The red arrow shows the position of the drainage tube. **d** Seventh-two hours after the STER surgery, a gastroscopy was performed to evaluate the esophageal wound. Simultaneously, the drainage tube was removed and the remaining wound was closed with a metal clip.

To verify that prophylactic wound drainage is capable of reducing the complications of esophageal fistula and infection following the STER operation, we continuously monitored the blood routine and measured white blood cell (WBC) levels postoperatively. It was discovered that prophylactic wound drainage could expedite return of WBC levels to normal ( Fig. 2 **a** ). Furthermore, the level of procalcitonin in plasma was significantly lower 48 hours after the operation than in the control group ( Fig. 2 **b** ). Simultaneously, we found that preventive drainage reduced fluid and gas accumulation under the esophageal wound following the STER procedure, which was demonstrated by chest CT examination 72 hours after the operation ( Fig. 2 **c** ). To further observe the esophageal wound, routine gastroscopy was performed 72 hours after the operation. It was discovered that preventive drainage remarkably decreased tissue necrosis and wound secretion ( Fig. 2 **d** ).

### STER postoperative esophageal fistula or infection is associated with impairment of the muscularis propria layer and mucosa

Previous investigations have verified that the dimensions, form, and original depth of esophageal submucosal tumors are elements contributing to risk of postoperative complications 7 8 9 . Larger tumors and those with irregular shapes may increase likelihood of intraoperative esophageal mucosal damage. Consequently, we examined the impact of mucosal and esophageal muscularis propria injuries on postoperative complications of STER. Intriguingly, despite our relatively small sample size, we discovered that mucosal and esophageal muscularis propria injuries significantly increased incidence of esophageal fistula and infectious complications following STER ( Table 2 ).

**Table:** **Table 2** Influence of different layers of esophageal injury on esophageal fistula or infection after STER.

Damage scope	Number of occurrences	Number of complications	Incidence
Submucosal layer	6	0	0%
Mucosal layer	7	6	85.71%
Muscularis propria layer	12	7	58.33%
Mucosal and Muscularis propria layer	6	6	100%
STER, submucosal tunneling endoscopic resection.			

## Discussion

STER is a revolutionary endoscopic technique that involves a minimally invasive preservation of mucosal integrity. Multiple clinical studies have demonstrated the safety and efficacy of STER for treatment of SMTs. In recent years, several studies have highlighted successful removal of giant esophageal leiomyomas utilizing either STER or piecemeal STER (P-STER), but these advancements have also been accompanied by reports of significant postoperative complications, including bleeding, esophageal fistula or hematocele, and infection 7 8 9 . In this paper, we conducted a comprehensive analysis of cases involving submucosal tumors of the esophagus with a transverse diameter exceeding 5 cm from 2020 to 2024. Notably, we observed a correlation between serious complications, including esophageal fistula and infection, and multiple factors related to surgical procedures. These factors include multiple ruptures of the esophageal mucosa, complete incision of the esophageal muscle layer, as well as postoperative bleeding. Currently, there is a notable deficiency in intraoperative prevention strategies aimed at mitigating risks of postoperative esophageal fistula or infection in patients undergoing STER for giant submucosal tumors in the esophagus, presenting with the aforementioned high-risk factors. Here, we have devised an ingenious approach by positioning the drainage device prior to securing the esophageal mucosa wound with metal clips. This innovative method effectively addresses the severe complications associated with esophageal fistula and infection, ensuring a more successful outcome.

Giant submucosal tumors of the esophagus mainly originate from the intrinsic muscle layer, and the most common pathological type is leiomyoma 10 11 . Giant leiomyomas in the esophagus exhibit a distinctive irregular shape. These tumors display a wide range of sizes and configurations, posing significant challenges in accurately predicting their appearance. The irregular morphology arises from the unregulated proliferation of smooth muscle cells, which have the capacity to disseminate uniformly within the esophageal wall 12 . Previous studies showed that the irregular shape of SMTs is a risk factor for STER-related major complications 6 13 . Endoscopic resection of irregular submucosal tumors is prone to damaging the esophageal mucosa, constituting the primary factor contributing to postoperative submucosal infections. Furthermore, resection of irregular submucosal tumors indeed necessitates longer surgery. The extended operative time inadvertently creates favorable conditions for translocation of esophageal colonization bacteria, potentially increasing risk of postoperative infections and complications. In addition, the giant leiomyoma in the esophagus, originating from the intrinsic muscle layer and exhibiting close and extensive continuity with this layer, poses a unique challenge during endoscopic resection. Because the entire muscle layer is detached during the procedure, a substantial cavity forms beneath the mucosa. Due to the inability of metal clips to fully close this large cavity, mucosal suturing can lead to favorable fluid accumulation and potential risk of bacterial infection.

Esophageal fistula or submucosal infection is the most severe STER-related complication that may require prompt attention and prevent further deterioration and be potentially life-threatening. Despite administering antibiotics post-STER surgery for removal of giant esophageal leiomyoma, we noted that a subset of patients exhibited severe complications, leading to a significant prolongation of their hospital stays 7 13 . A previous study confirmed that esophageal stent placement successfully diverted intraluminal contents from the tunnel, thus preventing contamination of the mediastinum and thorax 14 . However, utilization of metal stents has not only led to a substantial increase in hospitalization costs, but has also necessitated rehospitalization of patients for stent removal. In addition, it is worth noting that not all patients diagnosed with large esophageal leiomyomas will necessarily develop esophageal fistula or submucosal infection following STER surgery. Consequently, it is crucial to devise a straightforward prevention strategy that effectively addresses these clinical challenges and meets the evolving needs of patients.

Surgical drainage procedures have been widely employed as the primary approach for managing or averting postoperative infections, owing to their effectiveness in removing accumulated fluids and debris that can foster bacterial growth and subsequent infection 14 15 . To reduce submucosal infection following STER for giant esophageal leiomyoma, we placed a gastric tube under the wound mucosa for drainage prior to using metal clips for wound closure. Simultaneously, we observed the color and volume of the drainage fluid. After 48 hours, a routine endoscopic examination was performed. If no necrosis was observed at the wound surface, the drainage tube was immediately removed, and residual sinuses were closed with metal clips. Remarkably, none of our patients exhibited any symptoms indicative of esophageal fistula or submucosal infection. This favorable outcome underscores the effectiveness of our approach in mitigating risks associated with STER for giant esophageal leiomyoma.

Limitations of this study include the small sample size and absence of a randomized controlled trial (RCT). First, the surgical strategy necessitates involvement of additional professionals to offer precise assessments following its implementation, thereby facilitating determination of whether to proceed with its widespread adoption. Second, a multicenter RCT should be conducted to further confirm the safety and effectiveness of prophylactic drainage in reducing esophageal submucosal infection or perforation after endoscopic resection of giant esophageal submucosal tumors.

## Conclusions

Taken together, our study presents a novel strategy aimed at minimizing occurrence of esophageal fistula complications following STER surgery for giant esophageal submucosal tumors.
